# Response Surface Optimization of a Citric Acid Mechanochemical Alginate Extraction from *Laminaria digitata* for Fiber Production

**DOI:** 10.3390/polym18212683

**Published:** 2026-10-29

**Authors:** Kiri Belcher, Jan Tebben, Rebecca Möller, Laurie C. Hofmann

**Affiliations:** 1 Alfred Wegener Institute, Helmholtz Centre for Polar and Marine Research, Am Handelshafen, 27570 Bremerhaven, Germany; jan.tebben@awi.de (J.T.); laurie.c.hofmann@awi.de (L.C.H.); 2 University of Bremen, 28359 Bremen, Germany; 3 Faserinstitut Bremen e.V., Am Biologischen Garten 2, 28359 Bremen, Germany; moeller@faserinstitut.de; 4 Faculty of Technology, Bremerhaven University of Applied Sciences, An der Karlstadt 8, 27568 Bremerhaven, Germany

**Keywords:** alginate, mechanochemical, response surface methodology, wet spinning

## Abstract

Alginate fibers are a promising alternative to conventional synthetic fibers; however, the upstream process of extracting alginate remains energy and time intensive. This study aimed to optimize a mechanochemical alginate extraction method using a Box–Behnken design (BBD) and response surface methodology (RSM) and demonstrate its application through the wet spinning of alginate fibers and characterization of their properties. Acid extraction time, citric acid to algae loading ratio, and blade speed were evaluated against six responses: crude alginate yield, ash, phenolic content, protein content, intrinsic viscosity, and M/G ratio. Five responses show statistically significant models and were further investigated using Derringer’s desirability function identified the optimal conditions as 10 min acid extraction time, 4% loading ratio, and 1100 rpm blade speed. The optimized extraction design produced an 18.77% crude alginate yield, 9.01 dL g^−1^ intrinsic viscosity, 18.75% ash content, total phenolic content 10.07 mg GAE g^−1^, and M/G ratio of 1.99. The optimized alginate was successfully wet spun into continuous fibers. SEM images showed ribbed surface morphology and fiber diameter ranged from 182 to 269 µm. The tensile strength for the optimized alginate fibers averaged at 8.17 cN/tex and had a fineness of 472.6 dtex. Overall, the results show that a 10 min citric acid blade-based pretreatment can produce calcium alginate fibers from brown macroalgae.

## 1. Introduction

The growing demand for sustainable materials has increased interest in using biopolymers as a renewable alternative to petroleum-based products [1]. Within the framework of a circular bioeconomy, marine organisms are becoming a useful resource due to their high productivity, rapid renewability, and minimal use of land-based systems. Among the marine-derived biopolymers, alginate has received considerable attention because of its versatile functions and broad industrial applications [2].

Alginate is a linear copolymer composed of β-D-mannuronic (M) and α-L-guluronic (G) acid residues and is one of the most commercially valued biopolymers derived from brown macroalgae [3]. Due to its biocompatibility, gel-forming abilities, and adaptable physicochemical attributes, alginate is widely used in the food, pharmaceutical, biomedical, and textile industries [2]. The functional performance of alginate depends strongly on algal species, origin, as well as the harvest season and extraction conditions, which together influence key quality parameters such as yield, viscosity, molar mass, and block composition (M/G ratio) [4,5,6]. These properties, particularly the M/G ratio and viscosity, determine the suitability of alginate for specific industrial applications. Among these applications, alginate fibers have attracted increasing attention as sustainable alternatives to synthetic fibers [7]. The ability of alginate to be spun into fibers is strongly influenced by molecular weight, viscosity, and M/G composition [8]. Therefore, controlling extraction conditions is important not only for alginate yield but also for producing material suitable for fiber formation.

Conventional alginate extraction typically involves a mineral acid pretreatment, alkaline extraction, and alcohol precipitation, but these methods can produce environmentally harmful waste streams and cause polymer degradation [9,10]. Consequently, more sustainable extractions are being investigated and becoming well established [5]. The acid type and concentration strongly influence both extraction efficiency and alginate quality. While strong mineral acids such as hydrochloric acid can enhance polysaccharide release, they also present environmental and safety concerns and may promote depolymerization, reducing the molar mass and viscosity [10,11]. Citric acid has emerged as a promising food-grade alternative because it is less corrosive, safer to handle, and can achieve high alginate extraction yields and desirable polymer characteristics [12,13]. Considering that extraction conditions directly influence alginate physicochemical properties and quality; they play a crucial role in determining the spinnability and performance of biopolymer alginate fibers.

Alginate fibers, typically produced by wet-spinning methods, are increasingly considered as a sustainable and more biodegradable alternative to synthetic fibers, with growing interest driven by the expanding demand for natural-fiber applications [8]. The biocompatibility and high absorption qualities of alginate fibers have also supported biomedical uses, particularly in wound management [14], while broader alginate-based material applications have been reviewed in the context of bio-composites and functional biomaterials [7,15]. Fiber spinnability and end-use performance depend strongly on the molecular weight, M/G composition, and purity of alginate because these factors influence solution rheology and gelation behavior during spinning and crosslinking [7]. Therefore, when selecting an alginate extraction method for alginate fiber production, these parameters need to be considered. Ref. [8] demonstrated that a citric acid extraction of alginates from *Saccharina latissima* and *Laminaria digitata* can produce spinnable CaCl_2_-crosslinked fibers under their tested conditions. Following this, [16] spun fibers using the same protocol of a 30 min citric acid pretreatment followed by a 24 h sodium carbonate stage; however, long extraction durations even at a low agitation speed have been shown to lead to a high energy demand even when no heat is used [10]. Therefore, reducing the extraction time should be a critical extraction parameter to enable less energy usage. Despite these promising results, extraction optimization aimed specifically at reducing the extraction time and maintaining the alginate quality to produce consistent alginate fibers remains limited. Most studies have focused on maximizing alginate yield or characterizing extracted polymers, whereas the relationship between extraction conditions, alginate quality properties (e.g., molecular weight, M/G ratio, and impurity content), and following fiber spinnability remain poorly understood. Furthermore, sustainable extraction approaches designed specifically for alginate fiber production have received relatively little attention [5,7].

As a potential solution, mechanochemical pretreatment has emerged as a promising sustainable method, by the use of mechanical disruption, which reduces the need to use strong, hazardous chemicals and long extraction times, thereby lowering the energy demand [17]. It has already been shown to enable the citric acid pretreatment to be shortened to as little as 6 min without a loss of crude alginate yield or structural M/G changes and uses simple blade-based equipment that is already available and easier to scale up [12]. The use of higher blade speeds also improved alginate purity by reducing the ash and protein contents; however, higher blade speeds and longer pretreatment times depolymerized the alginate, as shown by a reducing molar mass [12]. Both purity and molar mass influence fiber spinnability [7], so both are important to consider for extraction methods. When combined with an organic acid such as citric acid, mechanochemical pretreatment may offer a more sustainable alternative to conventional mineral-acid routes, as it shortens the extraction time and avoids strong mineral acids while limiting polymer degradation and improving the purity of the extracted alginate [10,12].

Building on this approach, this study investigates a mechanochemical citric-acid pretreatment as a sustainable method to produce alginate suitable for being wet spun into calcium alginate fibers. Response surface methodology was used to optimize a mechanochemical citric-acid pretreatment for alginate extraction from *Laminaria digitata*. The effects of acid-to-algae ratio, blade rotational speed, and pretreatment time were evaluated in terms of crude yield, intrinsic viscosity, M/G ratio, and impurity levels (ash, protein, and phenolics). The aim of this work is to optimize the mechanochemical citric-acid pretreatment conditions to maximize alginate yield while minimizing extraction time and to assess the quality of the resulting alginate and its suitability for fiber formation. By simultaneously targeting high alginate yields and fiber relevant qualities, including molecular weight, M/G ratio, and impurity content, this study aims to support a more sustainable pathway for producing alginate fibers for scalable industrial applications.

## 2. Materials and Methods

### 2.1. Algae Collection and Preparation

*Laminaria digitata* fronds were collected in June 2024 by scientific SCUBA divers from the Alfred Wegener Institute Helmholtz Centre for Polar and Marine Research Center for Scientific Diving in the North Sea, near the coastal region of Helgoland, Germany (54°11′27.59″ N, 7°52′51.68″ E). The collected algae were washed with freshwater to remove any sand or debris. Excess water was drained, and then, they were freeze dried until a constant weight was achieved. The biomass was then ground using a Retsch GM 200 grinder mill (Retsch GmbH, Haan, Germany) and stored at −20 °C until further use in the extraction process.

### 2.2. Extraction of Alginate

Alginate extraction was performed according to [12], with modifications to the mechanochemical pretreatment stage and experimental design. The citric acid concentration (4% *w*:*v*), sodium carbonate concentration (2% *w*:*v*), and alkaline stage parameters (1:20 *w*:*v* algae to alkaline solution ratio, 200 rpm and 2 h duration) were held constant as described previously [12].

In summary, dried *L. digitata* biomass (18 g) was treated with a 4% citric acid concentration solution at three different acids: algae loading ratios (2%, 3%, and 4% i.e., 2, 3, and 4 g dried biomass per 100 mL of acid solution corresponding to 900, 600, and 450 mL) in a Thermomix (Vorwerk, Wuppertal, Germany) at different blade rotational speeds (200, 1100, and 2000 rpm) for three different times (10, 30, and 60 min). The pretreatment time was varied from a minimum of 10 min to a maximum of 60 min to enable sufficient ion exchange and to cover the lower and upper ends of alginate extraction times (30–60 min) [5]. No additional heat was used, and the temperature and pH were measured at the beginning using a WTW pH 3110 m (WTW, Weilheim, Germany) and the end of the acid extraction time and stayed constant at 2–2.5 pH and 20–22 °C, respectively.

Following the acid treatment, the biomass was removed, drained, and washed with Milli-Q water. The alkaline extraction was completed with a 2% sodium carbonate solution (1:20 *w*:*v*) at 200 rpm for 2 h in the Thermomix mixer. Subsequently, the biomass was strained using a muslin cloth; the supernatant was collected, then centrifuged at 3500 rpm for 20 min, and finally vacuum-filtered with perlite (VWR International GmbH, Darmstadt, Germany). The supernatant was collected and precipitated with 70% ethanol (1:2 *v*:*v*). The precipitated alginate was collected, rinsed very briefly with ultra-pure Milli-Q grade water to remove surface ethanol before freezing and then freeze-dried, and stored at 4 °C until further analysis.

The crude alginate yield was calculated using Equation (1):

Crude Alginate Yield (%) = ((Mass of dried crude alginate (g))/(Mass of dried biomass (g)) × 100(1)

### 2.3. Characterization of Impurities

#### 2.3.1. Ash Content

The ash content was determined according to [12] with some modifications. Approximately 2 g of alginate was placed in pre-weighed crucibles and combusted in a muffle furnace (Thermoncept, Bremen, Germany) at 800 °C. Following ignition, the crucible was cooled in a desiccator and weighed. The ignition, cooling, and weighing cycle was repeated until a constant mass was achieved. A constant mass was achieved after three ignition cycles (5 h each), corresponding to a cumulative ignition time of approximately 15 h.

The ash content was calculated gravimetrically using Equation (2):

Ash % = ((Weight after combustion (g) − weight of crucible (g))/(Total Dry matter (g))) × 100(2)

#### 2.3.2. Protein Assay

Protein contamination was quantified using a Bradford assay according to [12]. A 1% (*w*:*v*) alginate solution was prepared in distilled water and reacted with Coomassie Brilliant Blue dye. Absorbance was measured at 595 nm, and the protein concentration was determined using a bovine serum albumin (BSA) calibration curve.

#### 2.3.3. Folin-Ciocalteu Assay

The total phenolic content of alginate samples was determined using the Folin–Ciocalteu assay [16]. A sodium carbonate stock solution was prepared 24 h in advance by dissolving 20 g of sodium carbonate in 80 mL Milli-Q water under heating and magnetic stirring until boiling. The solution was cooled, transferred to a bottle, and allowed to sit for 24 h. Prior to use, the sodium carbonate solution was filtered through a syringe filter. Alginate samples were prepared at least one day in advance by dissolving 0.02 g of crude alginate in 2 mL Milli-Q water to obtain a 1% (*w*:*v*) solution. For the assay, 10 µL of alginate solution was pipetted into PMMA semi-micro disposable cuvettes (1.5 mL), followed by the addition of 790 µL Milli-Q water and 50 µL of Folin–Ciocalteu reagent. After 30 s and within 8 min, 150 µL of the filtered sodium carbonate stock solution was added and mixed thoroughly by pipetting. The reaction mixtures were incubated at 20 °C for 2 h in the dark, after which absorbance was measured at 765 nm against a blank using a spectrophotometer. Calibration was performed using gallic acid standards with concentrations ranging from 10 to 300 µg mL^−1^. The total phenolic content was expressed as mg of gallic acid equivalents (GAE) per gram of dry weight.

### 2.4. Intrinsic Viscosity Determination

Intrinsic viscosity measurements were determined according to [12]. Measurements were performed using a Ubbelohde capillary viscometer (Type 0a, Xylem Analytics Germany Sales GmbH & Co.KG (SI Analytics), Mainz, Germany) at 25 °C in 0.1 M NaCl.

The intrinsic viscosity was obtained by extrapolation of reduced viscosity (η_sp_/c) to zero concentration

[η] = lim(c→0) (η_sp_/c) (3)

The viscosity average molar mass (Mv) of the crude alginate was determined using the Mark–Houwink–Sakurada Equation, Equation (4):

[η] = K·Mv^a^ (4)

where K = 0.023 g dL^−1^ and α = 0.984 [18].

### 2.5. Structural Classification of the Crude Alginate

#### Block Distribution and NMR Analysis

The NMR-based block composition and M/G ratio were determined according to American Society for Testing and Materials (ASTM) Standard F2259-10, the standardized method for assessing the structural of alginate following the work of [19,20].

Briefly, alginate samples (30 mg) were hydrolyzed, neutralized, and lyophilized as described in [13]. The hydrolyzed dried sample was dissolved overnight in 600 µL deuterated D2O, and 20 μL of 0.3 M triethylenetetramine-hexaacetic acid (TTHA) was added as a chelator. Following centrifugation, 450 µL of the supernatant was transferred to 5 mm NMR tubes (Wilmad^®^, Vineland, NJ, USA), and 11 µL of 0.5% TSP was added as the internal chemical-shift reference. ^1^H NMR spectra were acquired at 348 K using a Bruker AVANCE Neo 600 MHz NMR spectrometer (Bruker BioSpin GmbH, Rheinstetten, Germany) equipped with a 5 mm CPTCI cryoprobe and standard Bruker pulse programs. Spectra were referenced to TSP (0 pm for ^1^H-NMR). The proton spectra were acquired using a 30° pulse, with ns = 128, aq = 5 s and d1 = 5 s. Spectra were processed using MestReNova 14.3.3 software, and block distribution parameters were calculated according to the ASTM method F2259-10. The fractional calculations and M/G ratio can be found in the Supplementary Data.

### 2.6. Experimental Design

A Box–Behnken experimental design (BBD), a form of response surface methodology (RSM), was used to analyze and optimize the impacts of mechanochemical pretreatment with citric acid on the functional and quality properties of alginate. Three independent variables were defined: blade rotational speed, time, and acid-to-algae loading ratio (*w*:*v*), each examined at three levels (200, 1100, and 2000 rpm; 10, 30, and 60 min; and 2%, 3%, and 4% acid: algae loading ratio, respectively) based on previous studies on citric acid-assisted alginate extraction (Table 1).

The response variables evaluated included crude alginate yield, intrinsic viscosity, mannuronic-to-guluronic acid (M/G) ratio, ash content, protein content, and phenolic content (Table 2.) The experimental design consisted of 12 experimental runs, including five replicates at the central point to assess experimental variability and ensure reproducibility.

The data was subjected to a second- order polynomial model (Equation (5)):

(5) $$Y\;=\;\beta_{0}\;+\;{\Sigma_{i=1}}^{k}\;\beta_{i}X_{i}\;+\;{\Sigma_{i=1}}^{k}\;\beta_{ii}{X_{i}}^{2}\;+\;{\Sigma_{i=1}}^{k-1}\;{\Sigma_{j=i+1}}^{k}\;\beta_{ij}X_{i}X_{j}\;+\;\varepsilon$$

where *Y* is the predicted response, *β*_0_ is the intercept term, *β*_i_, *β*_ii_, *β*_ij_, are the regression coefficients for the linear, quadratic, and interaction effects, and (*X*_i_, *X*_j_), are the independent variables (time, acid: algae loading ratio and blade speed) and ε, is the residual error.

### 2.7. Validation of Optimized Conditions and Predictive Models

To identify the optimal alginate extraction conditions using citric acid mechanochemical extraction, Derringer–Suich desirability function methodology was applied to simultaneously optimize multiple responses, including crude alginate yield, intrinsic viscosity, M/G ratio, and impurities [21]. After the models were developed, predicted response values were generated on this grid and converted to individual desirability scores ranging from 0 (undesirable) to 1 (fully desirable). Individual desirability functions were assigned to each response: crude yield and intrinsic viscosity were maximized, while ash and phenolic content were minimized. The M/G ratio was also minimized in accordance with the targeted fiber-forming properties of the alginate.

A composite desirability index was calculated as the geometric mean of individual desirability. The protein content was excluded from the primary optimization due to the lack of statistical significance of its fitted model across the experimental domain.

Once the optimum conditions were identified, model validation was performed by conducting five replicate experiments at the predicted optimum. Experimental response values were compared with model-predicted values to confirm the adequacy of the fitted models and the reliability of the predicted optimal operating conditions.

### 2.8. Wet Spinning of Alginate Fibers

Wet spinning was used to process the extracted alginate into Ca^2+^-crosslinked fibers for evaluation of their fiber-forming ability under controlled spinning conditions [7]. Once the optimized alginate extraction conditions were identified, they were tested for suitability for producing alginate fibers from the extracted alginate. The five replicates from the optimized conditions were then used to assess the fiber spinning properties. Out of the five batch alginate replicates (*n* = 5), one sample was lost during handling so only four fibers were spun reported as *n* = 4 for all fiber properties.

For fiber spinning, a 7 wt.% crude alginate solution, a lab-built wet spinning setup consisting of a syringe pump, a 70 g/L CaCl_2_ coagulation bath, and a motor-controlled winder were used. Although fiber properties are strongly influenced by processing parameters such as extrusion diameter, coagulation bath composition, extrusion speed, and winding speed, all spinning conditions were kept constant in the present study to ensure comparability between samples derived from the optimized replicates.

The spinning dopes were characterized in terms of solid concentration (wt.%), dynamic rheology at 40 °C, calculated fiber fineness (dtex), and tensile strength (cN/tex) for the 5 replicates tested. They were compared against four commercial sodium alginate standards from Thermo Fisher Scientific (Waltham, MA, USA; type: normal, low, and very low viscosity) and Carl Roth GmbH + Co. KG (Karlsruhe, Germany; type: normal viscosity).

Individual alginate solutions were injected into the CaCl_2_ (≥98% Carl Roth GmbH + Co. KG, Karlsruhe, Germany) bath at a fixed extrusion velocity of 340 mL/h using a 1.2 mm diameter needle. The fibers were continuously collected on a winding drum rotating at 1 revolution s^−1^. The circumference of the winding drum was 10.5 cm, corresponding to a winding velocity of 10.5 cm s^−1^. The resulting draw ratio was approximately 1.25, calculated as the ratio of the winding velocity to the extrusion velocity. The coagulation bath was 30 cm long, and the fiber passed through twice, giving an immersion path of 60 cm and Based on the winding velocity, the calculated coagulation time was approximately 5.7 s. After spinning, the fibers were washed for 2 h in a batch of (99% denatured bioethanol; Th. Geyer GmbH & Co. KG, Renningen, Germany) to wash away the unbound residual Ca^2+^ and Cl^−^ ions and prevent fiber adhesion and then air-dried for 12 h in a fume hood.

#### 2.8.1. Diameter Measurements

A 1 cm long sample was taken from each spun fiber at three different locations using a scanning electron microscope (SEM) Type EVO 10 from Carl Zeiss Microscopy GmbH, Jena, Germany with the software Smart SEM Version 6.05. The spun fibers were observed at an accelerating voltage of 15 kV and at magnifications of 27–461× to measure their diameter and to study their morphology.

#### 2.8.2. Linear Density and Tensile Properties

The linear density, reported as Fineness, of each fiber was determined with an automatic balance (Statimat 4U from Textechno, Herbert Stein GmbH & Co. KG, Monchengladbach, Germany) on a 10 m length of fiber at a feeding speed of 5 m/min. The tensile strength of the fibers was assessed with their in-house software Textechno34 Version 3.4.668 at 20 °C and 65% relative humidity. A 100 N load cell was used under a constant testing speed of 250 mm min^−1^. To perform these tests, ten fiber samples for each type of alginate were tested using a gauge length of 250 mm. The fibers were fixed in holding tabs to keep them in position. The lower clamp moves vertically downwards until the fiber breaks. The tensile strength and strain were then determined from the breaking point. Results are given as mean ± SE (*n* = 10 technical replicates).

### 2.9. Statistical Analysis

The Box–Behnken Design (BBD) and response surface methodology analysis were performed using R (version 4.4.1) with the rsm package (version 2.10.6) [22]. Multiple regression models were fitted to estimate linear, quadratic, and interaction effects of the process variables. Analysis of variance (ANOVA) was conducted within the same package to assess model significance (*p* < 0.05), and three-dimensional response surface plots were generated to visualize factor response relationships.

## 3. Results

To understand how mechanochemical pretreatment conditions influence crude alginate yield and fiber relevant qualities, a Box–Behnken response surface design was applied to model crude yield and functional characteristics. The experimental design matrix and corresponding responses are presented in Table 3.

### 3.1. Effect of Pretreatment Conditions on Crude Alginate Yield

Crude alginate yield varied significantly across the RSM Box–Behnken experimental design, ranging from 7.88% to 21.11% depending on pretreatment conditions (Table 3). The estimated regression coefficients for the quadratic response surface model are presented in Supplementary Table S1. Among the investigated factors, the acid-to-algae loading ratio was identified as the main factor that affected extraction yield, showing a highly significant linear effect (*p* < 0.001). In contrast, in this study, neither pretreatment time nor agitation speed showed significant linear contributions within the studied range, indicating that mechanical intensity and exposure duration alone were not sufficient to drive yield changes without sufficient acid availability.

**Figure 1:**
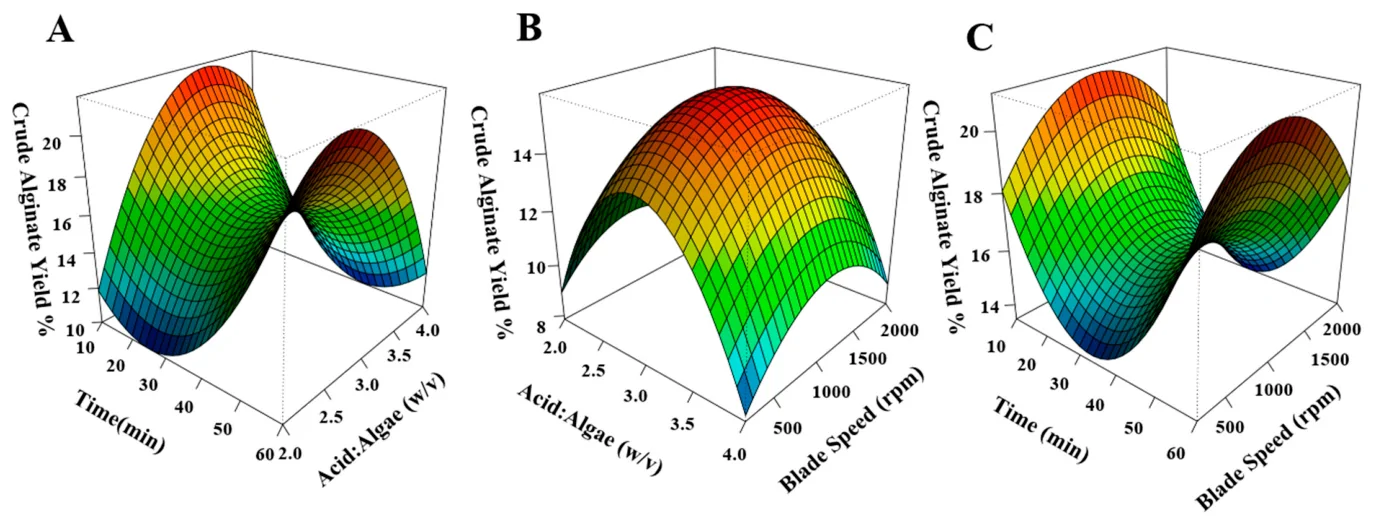
Three-dimensional response surface plots showing the interactive effects of pretreatment variables on crude alginate yield (%): (**A**) pretreatment time and acid-to-algae loading ratio at a fixed blade speed, (**B**) acid-to-algae loading ratio and agitation speed at a fixed pretreatment time, and (**C**) pretreatment time and blade rotational speed at a fixed acid-to-algae loading ratio. Surface colors represent the predicted crude alginate yield from the RSM model, with blue indicating lower yields and red indicating higher yields. The curved surfaces illustrate the quadratic relationships between process variables and the predicted extraction yield.

The response surface analysis further revealed a significant interaction between pretreatment time and acid concentration (*p* = 0.0016). Crude alginate yield increased with increasing acid-to-algae loading ratio, reaching a maximum at intermediate pretreatment times before declining slightly at longer treatment durations, indicating a quadratic response (Figure 1A). The pronounced curvature of the response surface confirms that excessive pretreatment time did not provide additional extraction benefits.

Similarly, Figure 1B demonstrates that the acid loading ratio affects crude yield across the range of blade speeds studied. The dome-shaped response surface suggests the existence of an optimum combination of acid loading and agitation speed, with reduced yields occurring at both lower and higher levels of these variables. Significant quadratic terms for time, acid concentration, and agitation speed indicated non-linear behavior and the presence of an optimum response region rather than a simple rise with increasing process intensity (Supplementary Table S3).

### 3.2. Effects of Pretreatment Conditions on Rheological Properties

Intrinsic viscosity ranged from 5.50 to 13.14 dL g^−1^ across the Box–Behnken experimental design (Table 3), indicating moderate variation in the rheological properties of the extracted alginates. However, the fitted second-order response surface model did not identify any statistically significant linear, interaction, or quadratic effects of pretreatment time, acid-to-algae loading ratio, or agitation speed on intrinsic viscosity within the investigated parameter range (*p* > 0.05 for all individual terms).

**Figure 2:**
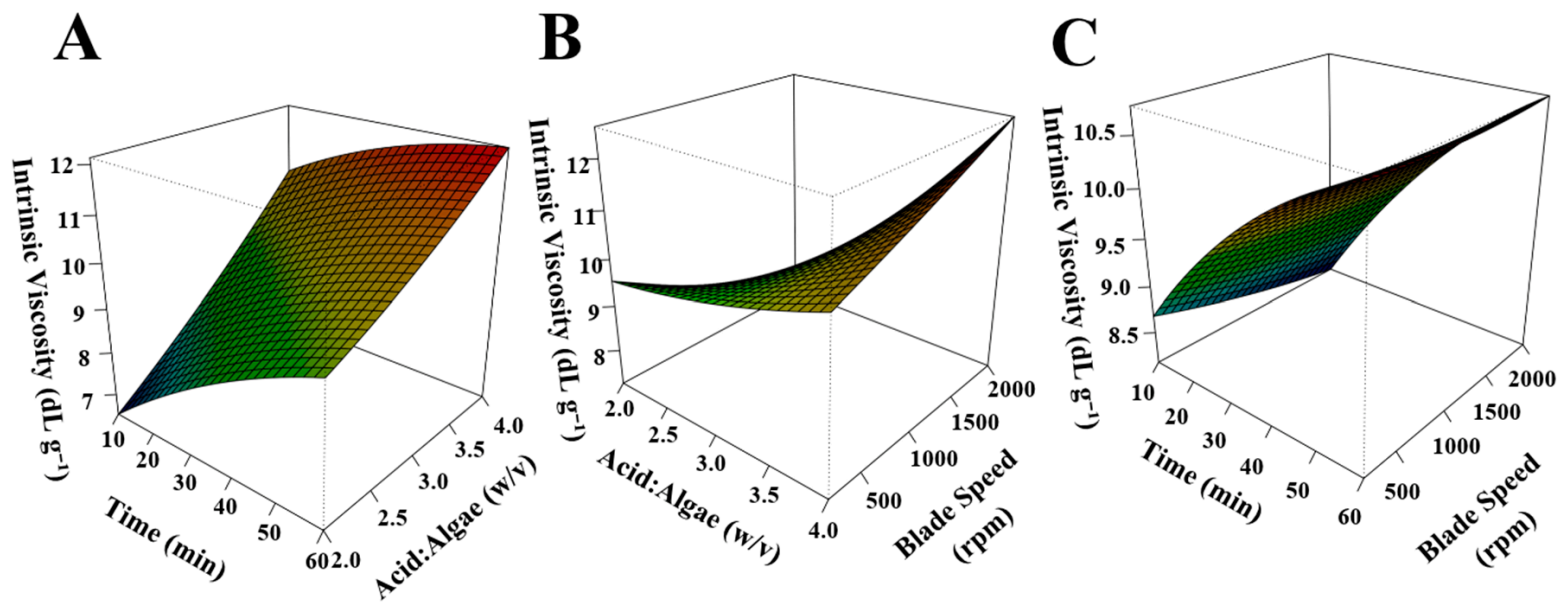
Three-dimensional response surface plots showing the interactive effects of pretreatment variables on intrinsic viscosity (dL g^−1^): (**A**) pretreatment time and acid-to-algae loading ratio at a fixed agitation speed, (**B**) acid-to-algae loading ratio and blade speed at a fixed pretreatment time, and (**C**) pretreatment time and blade rotational speed at a fixed acid-to-algae loading ratio. Surface colors represent the predicted intrinsic viscosity from the RSM model, with blue indicating lower values and red indicating higher values.

The fitted model was not statistically significant (F-test, *p* = 0.267), and there was a non-significant lack-of-fit (*p* = 0.435), indicating the model accurately represented the data despite the absence of any significant treatment effects. A near-stationary ridge condition was identified, with the calculated stationary point located partially outside the feasible experimental domain, including a non-physical negative rpm value (Figure 2).

### 3.3. Effects of Pretreatment Conditions on M/G Ratio

**Figure 3:**
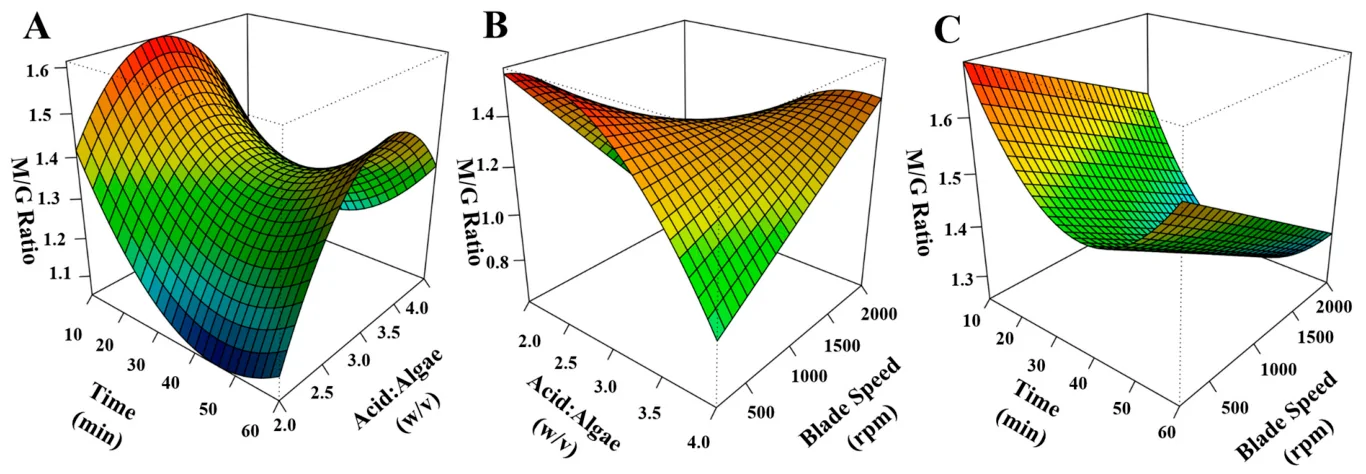
Three-dimensional response surface plots showing the interactive effects of pretreatment variables on M/G ratio: (**A**) pretreatment time and acid-to-algae loading ratio at a fixed blade speed, (**B**) acid-to-algae loading ratio and blade speed at a fixed pretreatment time, and (**C**) pretreatment time and blade rotational speed at a fixed acid-to-algae loading ratio. Surface colors represent the predicted M/G from the RSM model, with blue indicating lower values and red indicating higher values.

The M/G ratio was not significantly affected by pretreatment time, acid: algae loading ratio, or blade rotational speed (*p* = 0.70). None of the linear (*p* = 0.62), interaction (*p* = 0.77), or quadratic terms (*p* = 0.34) were statistically significant, indicating that the tested processing variables had limited influence on the mannuronic-to-guluronic acid composition of the extracted alginate (Figure 3). The response surface model showed a relatively poor predictive capability, with an R^2^ of 0.404 and an adjusted R^2^ of −0.192, suggesting that much of the variation in M/G ratio was not explained by the selected pretreatment factors.

### 3.4. Effects of Pretreatment Conditions on Impurities

**Figure 4:**
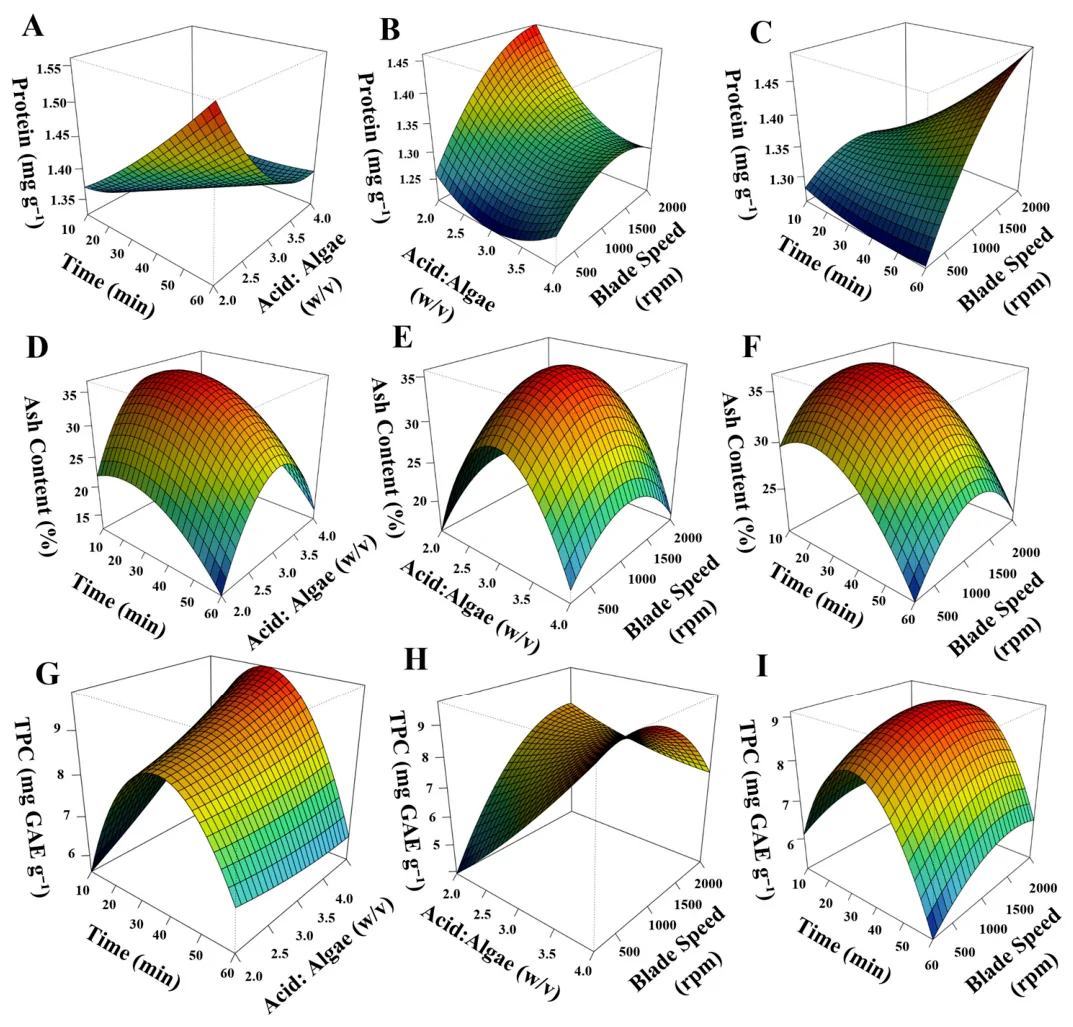
Three-dimensional response surface plots showing the interactive effects of pretreatment time (min), acid: algae loading ratio (*w*:*v*), and blade rotational speed (rpm) on crude alginate properties. (**A**–**C**) Protein (mg g^−1^), with (**A**) pretreatment time × acid concentration, (**B**) acid concentration × blade speed, and (**C**) pretreatment time × blade speed. (**D**–**F**) Ash content (%), with (**D**) pretreatment time × acid concentration, (**E**) acid concentration × blade speed, and (**F**) pretreatment time × blade speed. (**G**–**I**) Total phenolic content (mg GAE g^−1^), with (**G**) pretreatment time × acid concentration, (**H**) acid concentration × blade speed, and (**I**) pretreatment time × blade speed. Colours represent red being the highest recorded values and blue the lowest.

Impurity levels showed different responses to the pretreatment conditions across the Box–Behnken design. The protein content (Figure 4A) was not significantly affected by pretreatment time, acid-to-algae loading ratio, or blade rotational speed (*p* > 0.05), indicating that protein contamination remained relatively stable within the investigated processing range (Supplementary Tables S5–S7).

The quadratic response surface model for ash content was significant (*p* = 0.0085; R^2^ = 0.901), with acid concentration identified as the main contributing factor (Figure 4B). Both the linear and quadratic acid terms were significant (*p* < 0.05), and in addition, significant quadratic effects of pretreatment time and blade rotational speed suggested the presence of an optimum processing region for reducing mineral impurities (Supplementary Table S7).

The phenolic content was also significantly affected by the pretreatment conditions (*p* = 0.0181; R^2^ = 0.875). Pretreatment time had the strongest influence, showing both significant linear and quadratic effects (Supplementary Table S6). The response surface plots illustrated this trend, showing that intermediate pretreatment conditions produced lower phenolic contents compared to more higher processing combinations (Figure 4C).

### 3.5. Multi-Response Optimization and Validation Experiments

Multi-response optimization was conducted using the Derringer–Suich desirability function to identify pretreatment conditions that balance extraction performance, polymer quality, and impurity reduction [18]. To reduce the overall processing time and energy demand, the optimization was evaluated under a short-duration pretreatment condition (10 min). The composite desirability function identified an optimum at 10 min, 4.0% acid: algae concentration, and 1100 rpm, with an overall desirability of 0.66. Under these conditions, the fitted models predicted an ash content of 21.73%, crude alginate yield of 19.13%, intrinsic viscosity of 10.52 dL g^−1^, M/G ratio of 1.43, and total phenolic content of 8.17 mg GAE g^−1^ (Table 4).

Model validation was performed under the optimized conditions, with experimental values of 18.75 ± 0.60% ash content, 18.77 ± 3.03% yield, 9.01 ± 0.99 dL g^−1^ intrinsic viscosity, 1.99 ± 0.03 M/G ratio, and total phenolic content of 10.07 ± 1.07 mg GAE g^−1^ (Table 4). The predicted and experimental values showed good agreement for responses primarily associated with extraction performance and crude alginate characteristics.

### 3.6. Fiber Spinning Performance of Optimized Alginate vs. Commercial Standards

#### 3.6.1. Rheology

**Figure 5:**
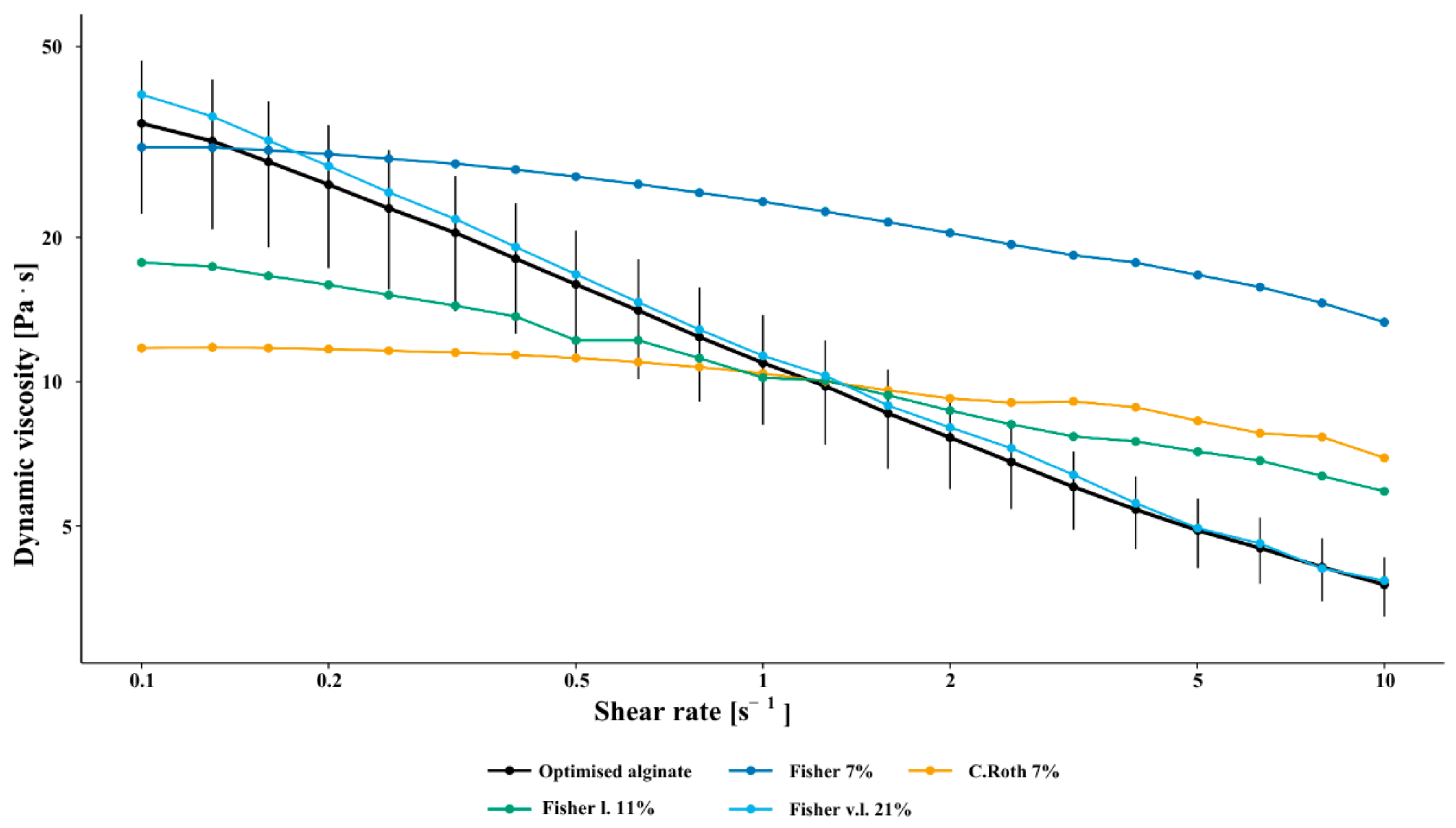
Dynamic viscosity as a function of shear rate for optimized and commercially available reference alginate solutions at 40 °C. The optimized alginate solution (7 wt.%) is presented as the mean across four independent extraction batches (*n* = 4), with error bars indicating ± standard error (SE). Commercially available reference alginates are shown as individual curves without error bars (*n* = 1). Both axes are displayed on logarithmic scales.

The dynamic viscosity of the optimised alginate solution (7 wt%) decreased with increasing shear rate, indicating shear-thinning behavior (Figure 5).

#### 3.6.2. Spinning Dope Properties and Processability

Wet-spinning trials using the optimized alginate (7 wt.%) demonstrated successful formation of the calcium alginate fibers. Out of the 5 batches that were produced from the optimized extraction methods, one sample was lost due to handling. The four resulting batches were successfully spun into fibers; the spinning dope exhibited a mean dynamic viscosity of 3.83 ± 0.39 Pa·s at 40 °C, enabling continuous extrusion and formation of uniform calcium alginate fibers. The resulting fibers showed a mean fineness of 472.5 ± 16.9 dtex, elongation break of 3.72 ± 0.94%, and tensile strength of 8.17 ± 0.72 cN/tex (Table 5).

Four commercial alginates were additionally spun in single replication to provide a reference material to the optimized extracted alginate. Standard-viscosity alginates, including C. Roth and Fisher normal grades were processed at 7 wt% solids and exhibited fineness values of 570 and 580 dtex, with tensile strengths of 5.9 and 8.7 cN/tex, respectively. The optimized alginate however produced finer fibers (472.5 dtex) but a similar tensile strength (8.17 cN/tex), indicating efficient fiber formation despite the lower fiber diameter.

The lower-viscosity Fisher alginates displayed substantially coarser fibers (1000 and 2000 dtex); however, these materials required increased polymer concentrations (11 and 21 wt%, respectively) to achieve stable spinning conditions. The elongation at break for the fibers was 1.5 ± 0.1% and 0.7%, respectively for the low- and very-low-viscosity Fisher alginates, and the normal-viscosity Fisher and C. Roth alginate fibers were at 5.0 ± 0.8% and 1.2 ± 0.1%.

The relationship between alginate molecular characteristics and fiber performance was further evaluated through intrinsic viscosity and M/G ratio measurements (Supplementary Table S5). The commercial alginate references showed M/G ratios between 1.12 and 1.20, whereas the optimized extracted alginate exhibited a higher M/G ratio of 1.99 ± 0.03.

The intrinsic viscosity of the optimized extracted alginate (9.01 ± 0.99 dL g^−1^) was similar to that of the spinnable C. Roth normal-viscosity alginate (9.86 dL g^−1^) and within the range associated with fiber-forming commercial materials. In contrast, the non-spinnable Thermo Fisher very-low-viscosity alginate exhibited a lower intrinsic viscosity (2.65 dL g^−1^), suggesting insufficient polymer chain length and reduced chain entanglement for effective fiber formation.

#### 3.6.3. Morphology and Scanning Electron Microscopy (SEM) of the Fibres

**Figure 6:**
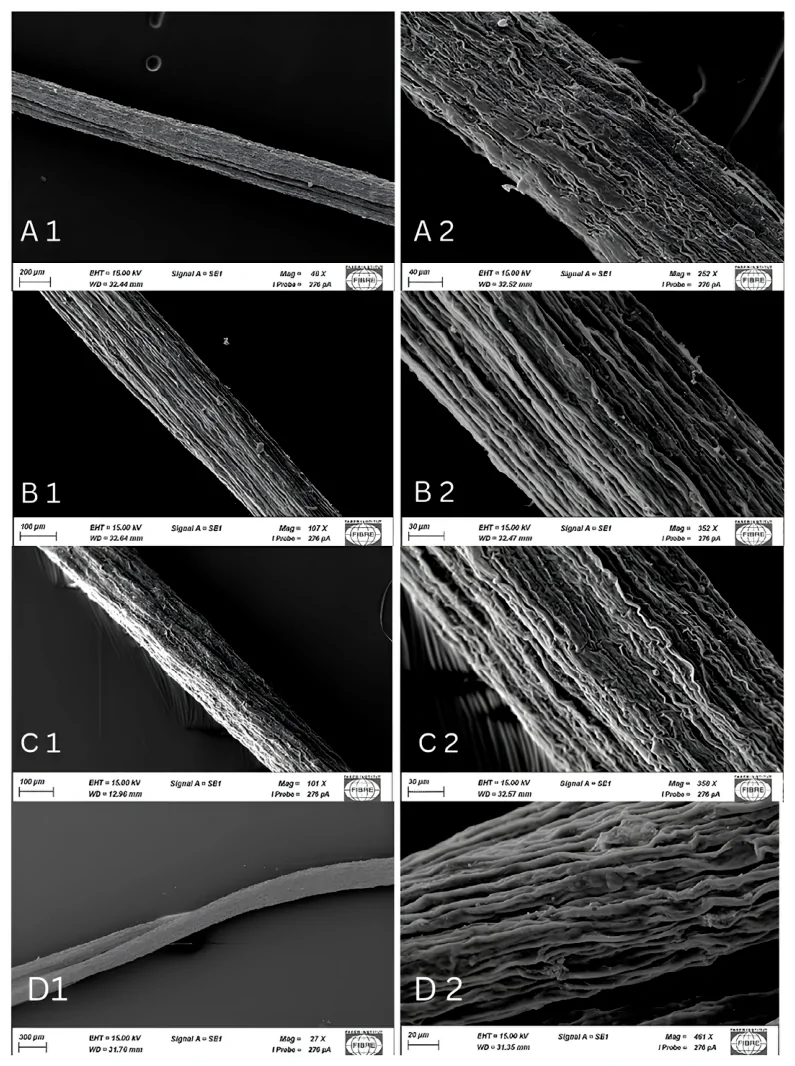
Scanning electron microscopy (SEM) images of wet-spun alginate fibers produced from optimized extraction replicates. Representative SEM micrographs showing the morphology and surface structure of fibers produced from (**A**) Sample 1, (**B**) Sample 2, (**C**) Sample 3, and (**D**) Sample 4. Low magnification images (**A1**–**D1**) show the overall fiber morphology, while higher magnification images (**A2**–**D2**) reveal surface features and fibrillar structure. Scale bars correspond to 100 µm for (**A1**–**D1**), 40 µm for (**A2**), 30 µm for (**B2**,**C2**), and 20 µm for (**D2**).

SEM imaging confirmed that all fiber samples exhibited a predominantly ribbed surface morphology typical of CaCl_2_ crosslinked alginate fibers (Figure 6). No significant structural differences were observed between samples, indicating consistent fiber formation across different extraction replicates.

**Figure 7:**
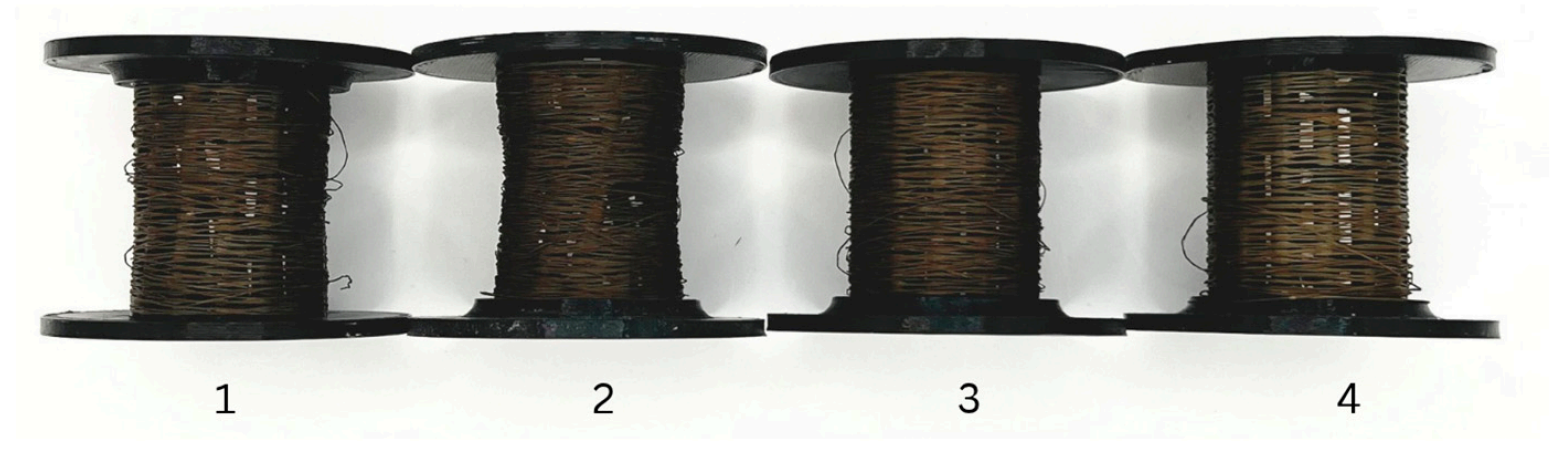
Spools of the optimized alginate fiber replicates (1–4).

Fiber morphology analysis revealed consistent structural features across all wet-spun alginate samples (Figure 7). Fiber diameter measurements ranged from 182 to 269 µm, depending on the sample (Supplementary Table S6). Sample 2 and Sample 3 produced comparatively finer fibers (201 µm and 182 µm, respectively), whereas Sample 1 and Sample 4 exhibited larger diameters (265 µm and 269 µm).

## 4. Discussion

### 4.1. Effects of Pretreatment Conditions on Crude Alginate Yield

Acid pretreatment within an alginate extraction promotes proton exchange with divalent cations, particularly calcium, converting insoluble calcium alginate within the cell wall matrix into alginic acid [2]. This demineralization step disrupts ionic cross-linking, weakens the structural integrity of the cell wall, and enhances the accessibility of sodium carbonate during the subsequent alkaline extraction, thereby facilitating alginate solubilization [9]. Crude alginate yield in this study varied significantly across the Box–Behnken experimental design, ranging from 7.88% to 21.11% depending on pretreatment conditions (Table 3). Crude alginate yield in this study was determined by gravimetric methods and the non-alginate components such as ash content, protein, and phenols are also reported to ensure yields are comparable across the literature [23]. The increasing values with increasing acid to algae loading ratio are consistent with those reported by [24], who demonstrated that the hydrochloric acid-to-algae ratio significantly influenced alginate yield during ultrasound-assisted acid pretreatment of *Laminaria digitata*. They identified the liquid-to-solid ratio as the most influential linear factor in their Box–Behnken design model and similarly observed a strong interaction with pretreatment time, with prolonged acid exposure resulting in diminishing improvements in alginate yield. However, in this study, the longer acid pretreatment time offered no further improvement in yield, suggesting that most of the readily accessible calcium had already been removed and that additional treatment provided diminishing returns.

Although the acid to algae loading ratio had an effect on alginate yield, neither pretreatment time nor blade speed showed significant linear contributions (Figure 1), suggesting that most of the readily accessible calcium ions had already been removed and that additional treatment provided diminishing returns. Overall, alginate yield was primarily influenced by acid availability within the pretreatment, while mechanical parameters played a secondary role through interaction and curvature effects (Figure 1C). These findings highlight that both acid availability and processing conditions should be taken into consideration in order to maximize polymer yield.

### 4.2. Effects of Pretreatment Conditions on Intrinsic Viscosity and M/G Ratio

A key indicator of the molecular size and chain confomation of alginates in solution is assessing the intrinsic viscosity of alginate (η), which reflects the hydrodynamic behavior of the polymer. It is defined by the Huggins equation. In this study, the intrinsic viscosity ranged from 5.50 to 13.14 dL g^−1^ across the Box–Behnken experimental design; however, the overall model was not statistically significant, which indicates that the observed variability was not due to major model inadequacy but more likely reflected experimental variation and/or additional factors not captured within the experimental design (Figure 2).

This finding is consistent with that of [13], who investigated ultrasound-assisted citric acid extraction of alginate from *Sargassum cymosum*. They reported that within the citric acid pretreatment, ultrasound power and treatment time had no significant effect on viscosity-average molecular weight (Mw). The absence of significant effects of pretreatment variables on intrinsic viscosity in the present study contrasts the recent findings from [12], who reported that increasing blade rotational speed from 200 to 2000 rpm significantly increased intrinsic viscosity following a 6 min citric acid pretreatment. They attributed this improvement to enhanced biomass disruption and more complete removal of impurities, resulting in alginate solutions that exhibited more ideal polyelectrolyte behavior at higher blade speeds. In contrast, the present Box–Behnken optimization did not identify any significant effects of the factors. This difference is likely explained by the narrower range of operating conditions examined in the current study, where the pretreatment conditions were already sufficient to achieve effective cell disruption without further altering the molecular characteristics of the extracted alginate. However, intrinsic viscosity may also be influenced by residual impurities, including salts and proteins, which can screen electrostatic interactions and restrict polymer chain expansion in solution [25]. Therefore, intrinsic viscosity should be interpreted with this in mind.

When looking at the structural properties of the alginate extracted, the M/G ratio also was not significantly affected by any of the tested parameters, the lack of significant effects indicates that the structural composition of alginate was largely preserved during the citric acid pretreatment process. Unlike yield or viscosity, which are strongly influenced by cell disruption, polymer solubilization, and molecular weight changes, the M/G ratio reflects the intrinsic distribution of mannuronic and guluronic acid residues within the alginate backbone [11]. This composition is primarily determined by the seaweed species and is generally less influenced or structurally changed under mild extraction conditions.

These findings are consistent with previous studies reporting minimal changes in alginate monomer composition following acid-assisted extraction. Refs. [12,13] similarly reported that citric acid pretreatment did not significantly alter M/G ratios under comparable extraction conditions. Furthermore, [11] observed only minor differences between alginates extracted using different acid systems, with citric acid producing alginate with comparable M/G characteristics to conventional mineral acid extraction.

The response surface plots showed no clear optimum region, with a relatively flat surface and variation occurring without a consistent trend across the experimental domain. This suggests that expanding the experimental range or incorporating additional biological factors, such as seasonal variation, species variability, or biomass composition, would likely be required to improve prediction of M/G ratio. Overall, the results demonstrate that the applied citric acid pretreatment conditions effectively enhanced alginate extraction while maintaining the native uronic acid composition of the polymer.

### 4.3. Effects of Pretreatment Conditions on Impurity Content

Quantifying co-extracted impurities within crude alginate yield is a key indicator of extraction performance because impurities affect both alginate yield and functional performance of the final extract. Crude alginate yields determined by gravimetric methods tend to be overestimated. Therefore, reporting the non-alginate components alongside alginate purity ensures that yields can be compared across separate studies [23].

Additionally, impurities also influence functional quality, as non-alginate material lowers the proportion of gelling polymer, residual salts and divalent cations can alter ionic strength, viscosity, and gelation, and co-extracted proteins and polyphenols can affect color, clarity, and rheology, with additional concerns about immunogenicity in biomedical use [26]. In this study, the impurity content showed different responses to the pretreatment conditions across the Box–Behnken design. When looking at the protein content (Figure 4A), there was no significant difference across the experimental range (*p* > 0.05), indicating that protein contamination remained relatively stable within the investigated processing range (Supplementary Tables S5–S7).

When interpreting the ash content, the food chemical codex specifies an ash content of around 18–27% (dry weight) for sodium alginate because sodium leaves approximately 27% residue (as NA_2_CO_3_) after combustion [27]. In this study, the ash content ranged from 16 to 18%, which was below this range (Table 3); however, some exceeded 36%. This could potentially be due to residual inorganic salts from the alkaline extraction stage that were not fully removed during washing and precipitation, as similar high ash contents (22–35%) have been reported for crude alginates [28,29]. The ash content followed a non-linear trend, indicating that ash removal depended strongly on the acid to algae loading ratio. In addition, significant quadratic effects of pretreatment time and blade rotational speed suggested the presence of an optimum processing region for reducing mineral impurities (Supplementary Table S7). These findings are consistent with the work of [30], who demonstrated that citrate-based extraction systems effectively remove mineral components through calcium chelation, resulting in alginate-rich soluble fractions with reduced residual ash. Their study further showed that greater availability of citrate enhanced calcium removal and alginate solubilization, highlighting the importance of the citrate-to-biomass ratio during extraction. Similarly, the significant effect of the acid-to-algae loading ratio observed in the present study suggests that increasing the amount of citric acid available per unit mass of algae improved mineral removal up to an optimum, beyond which further increases provided little additional benefit. The corresponding response surface plots further demonstrated the non-linear relationship between processing severity and ash reduction (Figure 4B).

The phenolic content was also significantly affected by the pretreatment conditions, time had the strongest influence, showing both significant linear and quadratic effects. This indicates that moderate processing times were more effective for reducing phenolic compounds than either very short or prolonged treatments (Supplementary Table S3). The response surface plots illustrated this trend, showing that intermediate pretreatment conditions produced lower phenolic contents compared to more extreme processing combinations (Figure 4C). These findings are consistent with the role of acid pretreatment in removing phenolic compounds prior to alkaline extraction. Acid treatment promotes the conversion of calcium alginate to alginic acid while facilitating phenolic leaching from the biomass [9]. This is important as residual phenolics can oxidize during alkaline extraction, forming pigments that reduce alginate purity [2]. Overall, the results are particularly relevant for fiber production, as lower impurity levels may contribute to improved spinning stability and more consistent fiber formation.

### 4.4. Validation Experiments

Model validation was performed under the optimized conditions (Table 4). The predicted and experimental values showed good agreement for responses primarily associated with extraction performance and crude alginate characteristics, demonstrating the suitability of the model for describing the effects of pretreatment conditions on alginate recovery. However, compositional parameters such as the ash content and M/G ratio showed greater variability. Unlike yield and viscosity, which are directly influenced by cell wall disruption, alginate solubilization, and polymer chain preservation, the M/G ratio represents the relative distribution of mannuronic and guluronic acid residues within the polymer backbone. This structural characteristic is largely determined by the biological origin of the alginate and the composition of the seaweed species, rather than being strongly modified by mild extraction conditions [2].

The limited variation in the M/G ratio across the experimental design indicates that the citric acid pretreatment was effective in increasing the alginate yield while maintaining the structural composition of the polymer, which is similar to results presented in [12]. Despite these differences, the low standard deviations indicate good experimental reproducibility. Overall, the results confirm that desirability-based optimization provides a robust framework for identifying suitable processing conditions, although compositional parameters may require further model refinement [21].

### 4.5. Fiber Spinning Performance

When assessing fiber spinning performance, there are several factors that affect the final alginate fiber formation and following mechanical properties. Firstly, when using wet spinning as a technique, fiber morphology depends on the alginate to CaCl_2_ concentration ratio and the coagulation time [31]. The optimized alginate (7 wt.%) here had a mean dynamic viscosity of 3.83 ± 0.39 Pa·s at 40 °C, which allowed continuous extrusion and uniform fiber formation into a 70 g L^−1^ calcium chloride concentration. This low dynamic viscosity could be due to the high ash content (18.75%) of the optimized samples, thereby lowering the actual dope concentration. The resulting fibers had a mean fineness of 472.5 ± 16.9 dtex, a tensile strength of 8.17 ± 0.72 cN/tex, and an elongation at break of 3.72 ± 0.94% (Table 5).

When looking at other studies on alginate fiber formation, ref. [31] varied these parameters and identified optimal conditions of 8 wt.% alginate, 1 wt.% CaCl_2_, and a 5 min coagulation time; the fibers produced had an average tenacity of 11 cN/tex and an average elongation at break of 24%. Although there was a similar alginate concentration, in this study, a higher calcium chloride coagulation concentration was used (70 g L^−1^; 7%), and [31] found that a higher CaCl_2_ concentration increased cross-linking, which increased the stiffness while reducing elongation at break. When looking at the referenced material spun in the same concentration coagulation bath, for the low- and very-low-viscosity Fisher alginates, which were at an alginate concentration of 11 and 21%, were lower at 1.5 ± 0.1% and 0.7%. However, the normal-viscosity Fisher and C. Roth alginate fibers at a concentration of 7% had higher elongation breaks at 5.0 ± 0.8% and 1.2 ± 0.1%, and this could be due to the physiochemical properties of the alginates.

Fiber mechanical performance is strongly influenced by alginate molecular characteristics, particularly molecular weight and chain integrity, as longer polymer chains promote increased entanglement and more efficient stress transfer within the calcium alginate network [8]. The intrinsic viscosity in this study was 9.01 ± 0.99 d L g^−1^, which corresponds to a viscosity average molar mass of around 450 kDa. Ref. [16] used a commercial alginate with a 107.4 kDa and achieved successful fiber formation. The molar mass is generally associated with greater dope viscosity and better filament control during gel formation [32], although the two values are only comparable if similar Mark–Houwink parameters were used. The referenced materials had intrinsic visocisties of 2–13.56 dL g^−1^ (Supplementary Table S8), with the lowest viscosity Fisher alginate producing very low elongation break at 0.7% and 2.5 tensile strength (Table 5).

The relationship between alginate molecular characteristics and fiber performance was further evaluated through intrinsic viscosity, molar mass, and M/G ratio measurements (Supplementary Table S6). The commercial alginate references showed M/G ratios between 1.12 and 1.20, whereas the optimized extracted alginate exhibited a higher M/G ratio of 1.99 ± 0.03. A higher M/G ratio indicates alginate with a higher mannuronic acid content, which can be seen in Supplementary Table S9, where the F_m_ fraction was 0.67 and higher than the 0.2 F_G_ fraction. Mannuronic-rich alginates are known to form softer gels in comparison to higher guluronic acid alginates, which form stronger gels due to the carboxylate group on the alginate backbone [33].

Despite this difference in uronic acid composition, the extracted alginate demonstrated successful wet-spinning performance, indicating that a higher mannuronic acid proportion did not prevent calcium alginate fiber formation under the applied spinning conditions. These results indicate that maintaining the intrinsic viscosity and chain length were more critical for fiber formation than achieving a specific M/G ratio. Similar work demonstrating successful calcium alginate fiber spinning from high-M/G alginates extracted from *Saccharina latissima* and *Laminaria digitata* showed that spinnable alginates exhibited M/G ratios of 1.98–2.23 [8]. These findings further indicate that the M/G ratio alone does not determine fiber formation but rather that a sufficient molecular weight and chain integrity are critical for producing continuous fibers.

The morphology of the fibers was highly consistent across the optimized samples. A minor longitudinal fold was observed in Sample 4; however, this feature is attributed to processing-induced deformation during spinning rather than intrinsic material heterogeneity, as similar but less pronounced features were observed across other samples (Figure 6). Despite this variation in diameter, all fibers maintained their structural integrity during spinning, suggesting that the optimized alginate extracts provide stable processing behavior under fixed spinning conditions. The relatively narrow range of diameters further supports the use of these extraction parameters in producing spinnable alginate solutions suitable for fiber formation.

Overall, these results demonstrate that the optimized citric-acid mechanochemical extraction process produced alginate capable of forming alginate fibers at a relatively low polymer concentration. The ability to obtain comparable tensile performance to commercial fiber-grade alginates indicates that the mild extraction approach minimized polymer degradation while improving extraction efficiency, supporting the suitability of the extracted alginate for high-value fiber applications.

## 5. Conclusions

This study demonstrated the successful optimization of mechanochemical citric acid-assisted alginate extraction from *Laminaria digitata* using a Box–Behnken response surface design. The acid-to-algae ratio was identified as the main factor influencing crude alginate yield, while the treatment conditions maintained the intrinsic viscosity and M/G ratio parameters. The protein content showed no change due to the conditions tested. The optimal extraction conditions were identified as 10 min acid treatment time, a 4.0% acid-to-algae ratio, and a blade speed of 1100 rpm, providing a balance between crude yield, quality, and impurity reduction. Validation experiments confirmed good process reproducibility, and the alginate produced using the optimized process was assessed for its suitability for wet fiber spinning. The alginate from the optimized extraction produced continuous calcium crosslinked fibers with an average tensile strength of 8.17 ± 0.72 cN/tex and an elongation at break of 3.72 ± 0.94%, despite its relatively high mannuronic-rich M/G ratio of 1.99 ± 0.03. Overall, the results highlight the potential of rapid mechanochemical citric acid pretreatment as an efficient approach for producing calcium alginate fibers from brown seaweed. Future work should investigate the scalability of this method. Additionally, future work should include a life cycle assessment of the pilot scale process to quantify resource and energy requirements, evaluate alternative organic acids, and further optimize spinning conditions to improve fiber properties.

## Figures and Tables

**Table 1:** Factors and their coded (−1, 0, +1) and actual levels used in the Box–Behnken design. The coded levels represent low, central, and high values of each factor used in the Box–Behnken design.

Input Variables	Levels		
	−1	0	1
Time (min)	10	30	60
Acid: Algae (*w*:*v*%)	2	3	4
Blade Rotational speed (rpm)	200	1100	2000

**Table 2:** The implemented Box–Behnken design showing independent variables and coded levels.

Experiment	A: Time (Min)	B: Acid: Algae (*w*:*v*%)	C: Blade Speed (rpm)
1	(−1) 10	(−1) 2%	(0) 1100
2	(1) 60	(−1) 2%	(0) 1100
3	(−1) 10	(1) 4%	(0) 1100
4	(1) 60	(1) 4%	(0) 1100
5	(−1) 10	(0) 3%	(−1) 200
6	(1) 60	(0) 3%	(−1) 200
7	(−1) 10	(0) 3%	(1) 2000
8	(1) 60	(0) 3%	(1) 2000
9	(0) 30	(−1) 2%	(−1) 200
10	(0) 30	(1) 4%	(−1) 200
11	(0) 30	(−1) 2%	(1) 2000
12	(0) 30	(1) 4%	(1) 2000
C (a)	(0) 30	(0) 3%	(0) 1100

(a) Mean Center point from 5 replicates.

**Table 3:** The implemented Box–Behnken design for the independent variables (time, acid: algae, blade speed) and their responses (crude alginate yield, intrinsic viscosity, M/G ratio, ash content, protein and phenolic contents).

Experiment	A: Time (Min)	B: Acid/ Algae	C: Speed (rpm)	Crude Yield (%)	Intrinsic Viscosity (dL g^−1^)	M/G Ratio	Protein (mg g^−1^)	Ash Content (%)	Total Phenolic Content (mg GAE g^−1^)
1	(−1) 10	(−1) 2%	(0) 1100	13.33	5.5	1.17	1.34	17.87	6.68
2	(1) 60	(−1) 2%	(0) 1100	18.72	10.61	1.06	1.72	16.54	6.78
3	(−1) 10	(1) 4%	(0) 1100	20.61	9.55	1.35	1.22	17.57	7.98
4	(1) 60	(1) 4%	(0) 1100	10.55	13.14	1.59	1.41	18.16	5.08
5	(−1) 10	(0) 3%	(−1) 200	17.22	9.55	1.65	1.36	33.16	5.88
6	(1) 60	(0) 3%	(−1) 200	21.11	9.57	1.34	1.15	15.62	5.88
7	(−1) 10	(0) 3%	(1) 2000	17.33	9.32	1.77	1.39	33.06	5.48
8	(1) 60	(0) 3%	(1) 2000	18.88	9.9	1.39	1.41	17.48	6.48
9	(0) 30	(−1) 2%	(−1) 200	8.33	9.69	1.60	1.26	16.31	6.08
10	(0) 30	(1) 4%	(−1) 200	7.88	10.92	1.79	1.39	18.06	10.08
11	(0) 30	(−1) 2%	(1) 2000	8.05	7.08	1.13	1.43	16.36	8.78
12	(0) 30	(1) 4%	(1) 2000	9.27	12.45	1.43	1.39	16.32	9.48
C (a)	(0) 30	(0) 3%	(0) 1100	17.22	7.03	1.36	1.36	35.73	9.08

(a) Mean Center point from 5 replicates.

**Table 4:** Model validation results comparing experimental and predicted values for key responses under optimized conditions (*n* = 5).

Response	Experimental Result	Predicted Value
Ash content (%)	18.75 ± 0.60	21.73
Yield (%)	18.77 ± 3.03	19.13
Intrinsic viscosity (dL g^−1^)	9.01 ± 0.99	10.52
M/G ratio	1.99 ± 0.03	1.43
Total phenolic content (mg GAE g^−1^)	10.07 ± 1.07	8.17

**Table 5:** Mechanical performance and fineness comparison between optimized alginate and commercial alginates.

Material	Solid Concentration (*w*:*v*%)	Technical Replicates	Fineness (dtex)	Elongation at Break (%)	Tensile Strength (cN/tex)
Fisher (normal)	7%	20	580	5.0 ± 0.8	8.4 ± 0.5
C. Roth (normal)	7%	20	570	1.2 ± 0.1	5.9 ± 0.6
Fisher (low viscosity)	11%	20	1000	1.5 ± 0.1	5.7 ± 0.4
Fisher (very low viscosity)	21%	2	2000	0.7	2.5
Optimized extracted alginate	7%	10 per batch	472.5 ± 16.9	3.72 ± 0.94	8.17 ± 0.72

Values are reported as mean ± SE. For the commercial alginates, technical replicates represent individual fibers tested under the same spinning conditions. For the optimized extracted alginate, four independently produced spinning batches were evaluated, with up to 10 individual fiber samples tested per batch. Elongation at break is reported as the mean of the four-batch means ± SE between batches (*n* = 4 independent batches). The Fisher very-low-viscosity sample was tested using two technical replicates (*n* = 2), and no SE is reported.

## Data Availability

The data presented in this study are available on request from the corresponding author.

## Supplementary Materials

The following supporting information can be downloaded at: https://www.mdpi.com/article/10.3390/polym18212683/s1. Figure S1: Stacked ^1^H NMR spectra of the optimized extracted alginate (ALG) replicate four and the four commercial alginate standards. Spectra are shown in the anomeric proton region (approximately 4.35–5.15 ppm) and were used to determine the mannuronic acid to guluronic acid (M/G) ratio based on the characteristic resonances of the alginate monomer units; Table S1: BBD experimental design and measured responses for the citric acid mechanochemical extraction of Laminaria digitata alginate, including extraction time, acid-to-algae ratio and blade speed, crude alginate yield, intrinsic viscosity, viscosity-average molar mass, mannuronic-to-guluronic acid ratio (M/G), protein content, ash content, total phenolic content; Table S2: M/G ratio and individual block fractions of the alginate extracted from each experiment in the BBD experimental design; Table S3. Regression coefficients for the quadratic RSM model of alginate yield; Table S4. Regression coefficients for the quadratic RSM model of M/G ratio; Table S5. Regression coefficients for the quadratic RSM model of Protein (mg g^−1^); Table S6. Regression coefficients for the quadratic RSM model of Total phenolic content (mg GAE g^−1^); Table S7. Regression coefficients for the quadratic RSM model of Ash (%); Table S8. Intrinsic viscosity (dL g^−1^) and viscosity average molar mass (kDa) for the referenced material and optimized crude alginate. Commercial alginate values represent single measurements, while optimized extracted alginate values represent mean ± standard deviation (*n* = 5); Table S9: Fractional and block sequence distribution of the optimized extracted alginate and commercially available alginate references, including the mannuronic-to-guluronic acid ratio (M/G) and the relative diad and triads of the FG, FM, FGG, FMM, FGM, FGGG, FMGM and FGGM sequence motifs. Values are presented as mean ± SE where applicable; Table S10. Fiber diameter of wet-spun alginate fibers from optimized samples.

## References

[1] Edo G.I., Ndudi W., Ali A.B.M., Yousif E., Jikah A.N., Isoje E.F., Igbuku U.A., Mafe A.N., Opiti R.A., Madueke C.J. (2025). Biopolymers: An Inclusive Review. Hybrid. Adv..

[2] Abdul Khalil H.P.S., Lai T.K., Tye Y.Y., Rizal S., Chong E.W.N., Yap S.W., Hamzah A.A., Nurul Fazita M.R., Paridah M.T. (2018). A Review of Extractions of Seaweed Hydrocolloids: Properties and Applications. Express Polym. Lett..

[3] Kumbar S.G., Laurencin C.T., Deng M. (2014). Natural and Synthetic Biomedical Polymers.

[4] Hernández-Carmona G., Freile-Pelegrín Y., Hernández-Garibay E. (2013). Conventional and Alternative Technologies for the Extraction of Algal Polysaccharides. Functional Ingredients from Algae for Foods and Nutraceuticals.

[5] Saji S., Hebden A., Goswami P., Du C. (2022). A Brief Review on the Development of Alginate Extraction Process and Its Sustainability. Sustainability.

[6] Dobrinčić A., Balbino S., Zorić Z., Pedisić S., Bursać Kovačević D., Elez Garofulić I., Dragović-Uzelac V. (2020). Advanced Technologies for the Extraction of Marine Brown Algal Polysaccharides. Mar. Drugs.

[7] Qosim N., Dai Y., Williams G.R., Edirisinghe M. (2024). Structure, Properties, Forming, and Applications of Alginate Fibers: A Review. Int. Mater. Rev..

[8] Silva M.P., Badruddin I.J., Tonon T., Rahatekar S., Gomez L.D. (2023). Environmentally Benign Alginate Extraction and Fibres Spinning from Different European Brown Algae Species. Int. J. Biol. Macromol..

[9] Arvizu-Higuera D., Hernández-Carmona G., Rodríguez-Montesinos Y. (1995). Batch and Continuous Flow Systems during the Acid Pre-Extraction Stage in the Alginate Extraction Process. Cienc. Mar..

[10] Smith H.A., Cabling L.P.B., Leonard N.A., Dubrawski K.L., Buckley H.L. (2024). Green Alginate Extraction from Macrocystis Pyrifera for Bioplastic Applications: Physicochemical, Environmental Impact, and Chemical Hazard Analyses. ACS Sustain. Resour. Manag..

[11] John Kennedy J.P.K., Muthuramalingam J.B., Balasubramanian V.K., Balakrishnan M., Murugan K., Soorangkattan S., Nalluchamy K.D., Muniyasamy S., Murugan S., Khumalo M.V. (2024). The Production and Characterization of Alginate from Sargassum Wightii Greville Ex J. Agardh.: Exploring Diverse Acid Treatments and Analytical Techniques. Process Saf. Environ. Prot..

[12] Belcher K., Traeger F., Primpke S., Hofmann L.C. (2026). Rapid Organic Acid Mechanochemical Extraction of Alginate from *Laminaria digitata*. Bioresour. Technol..

[13] Chica L.R., Yamashita C., Nunes N.S.S., Negreiros A.T., Moraes I.C.F., Ferreira A.G., Mayer C.R.M., Haminiuk C.W.I., Branco C.C.Z., Branco I.G. (2024). Optimizing Alginate Extraction Using Box-Behnken Design: Improving Yield and Antioxidant Properties through Ultrasound-Assisted Citric Acid Extraction. Food Chem. Adv..

[14] Qin Y. (2008). The Gel Swelling Properties of Alginate Fibers and Their Applications in Wound Management. Polym. Adv. Technol..

[15] Zdiri K., Cayla A., Elamri A., Erard A., Salaun F. (2022). Alginate-Based Bio-Composites and Their Potential Applications. J. Funct. Biomater..

[16] Badruddin I.J., Silva M.P., Tonon T., Gomez L.D., Rahatekar S.S. (2024). Modulating the Properties of Brown Alga Alginate-Based Fibers Using Natural Cross-Linkers for Sustainable Textile and Fashion Applications. ACS Omega.

[17] Wu K., Ju T., Deng Y., Xi J. (2017). Mechanochemical Assisted Extraction: A Novel, Efficient, Eco-Friendly Technology. Trends Food Sci. Technol..

[18] Clementi F., Mancini M., Moresi M. (1998). Rheology of Alginate from Azotobacter Vinelandii in Aqueous Dispersions. J. Food Eng..

[19] Grasdalen H. (1983). High-Field, ^1^H-N.M.R. Spectroscopy of Alginate: Sequential Structure and Linkage Conformations. Carbohydr. Res..

[20] Grasdalen H., Larsen B., Smidsrød O. (1979). A p.m.r. Study of the Composition and Sequence of Uronate Residues in Alginates. Carbohydr. Res..

[21] Derringer G., Suich R. (1980). Simultaneous Optimization of Several Response Variables. J. Qual. Technol..

[22] Lenth R.V. (2009). Response-Surface Methods in *R*, Using Rsm. J. Stat. Softw..

[23] Nøkling-Eide K., Langeng A.M., Åslund A., Aachmann F.L., Sletta H., Arlov Ø. (2023). An Assessment of Physical and Chemical Conditions in Alginate Extraction from Two Cultivated Brown Algal Species in Norway: Alaria Esculenta and Saccharina Latissima. Algal Res..

[24] Zhu X., Healy L., Wanigasekara J., Zhao M., Padamati R.B., Karuppusamy S., Curtin J.F., Sivagnanam S.P., Rai D.K., Sun D.W. (2023). Characterisation of Laminarin Extracted from Brown Seaweed *Laminaria Digitata*, Using Optimized Ultrasound- and Ultrafiltration-Assisted Extraction Method. Algal Res..

[25] Smidsrød O. (1970). Solution Properties of Alginate. Carbohydr. Res..

[26] Dusseault J., Tam S.K., Ménard M., Polizu S., Jourdan G., Yahia L., Hallé J.P. (2006). Evaluation of Alginate Purification Methods: Effect on Polyphenol, Endotoxin, and Protein Contamination. J. Biomed. Mater. Res. A.

[27] USP (1981). Food Chemicals Codex.

[28] Benslima A., Sellimi S., Hamdi M., Nasri R., Jridi M., Cot D., Li S., Nasri M., Zouari N. (2021). The Brown Seaweed Cystoseira Schiffneri as a Source of Sodium Alginate: Chemical and Structural Characterization, and Antioxidant Activities. Food Biosci..

[29] Pasanda O.S.R., Azis A. (2018). The extraction of brown algae (Sargassum Sp) through calcium path to produce sodium alginate. J. Bahan Alam Terbarukan.

[30] Sterner M., Edlund U. (2016). Multicomponent Fractionation of Saccharina Latissima Brown Algae Using Chelating Salt Solutions. J. Appl. Phycol..

[31] Erard A., Aurélie C., Salaün F. Effects of the alginate and CACL2 concentrations imparting mechanical and morphological properties of wet spun filaments. Proceedings of the 21st World Textile Conference AUTEX 2022.

[32] Var C., Palamutcu S. (2024). Diverse Approaches in Wet-Spun Alginate Filament Production from the Textile Industry Perspective: From Process Optimization to Composite Filament Production. Polymers.

[33] Haug A., Larsen B., Smidsrød O. (1974). Uronic Acid Sequence in Alginate from Different Sources. Carbohydr. Res..

