# Supplementary material for: Response Surface Optimization of a Citric Acid Mechanochemical Alginate Extraction from Laminaria digitata for Fiber Production
Source: Polymers (Basel). 2026 Oct 29;18(21):2683. doi: 10.3390/polym18212683 (PMC13765953; doi:10.3390/polym18212683)
Supplement: Supplementary file 1 [file polymers-18-02683-s001.zip › polymers-4617682-supplementary.pdf]

Supplementary Material

NMR analysis Calculations

The alginate extracts were analysed according to the ASTM Standard F2259-10 standard test method for determining the chemical composition and sequential parameters of alginate via NMR spectroscopy (ASTM Standard F2259-10, 2012), as previously described (Grasdalen, 1983; Grasdalen et al., 1981). Spectra were processed using MestReNova 14.3.3 software and were calculated as follows:

$G = 0.5 \times (IA + IC + 0.5 \times (B1 + B2 + B3))$
$M = B4 + 0.5 \times (B1 + B2 + B3)$

M / G ratio = M / G

$GG = 0.5 \times (IA + IC - 0.5 \times (B1 + B2 + B3))$
$MG = GM = 0.5 \times (B1 + B2 + B3)$
$MM = B4$

$GGM = MGG = B1 \times 0.5 \times (B1 + B2 + B3) / (B1 + B2)$
$MMG = B2 \times 0.5 \times (B1 + B2 + B3) / (B1 + B2)$
$GGG = GG - GGM$

Sequence fraction information was then calculated as follows:

$F_{G} = G / (M + G)$

$F_{M} = M / (M + G)$

$F_{GG} = GG / (M + G)$

$F_{MM} = MM / (M + G)$

$F_{GM} = F_{MG} = MG / (M + G)$

$F_{GGG} = GGG / (M + G)$

$F_{MGM} = MGM / (M + G)$

$F_{GGM} = F_{MGG} = GGM / (M + G)$

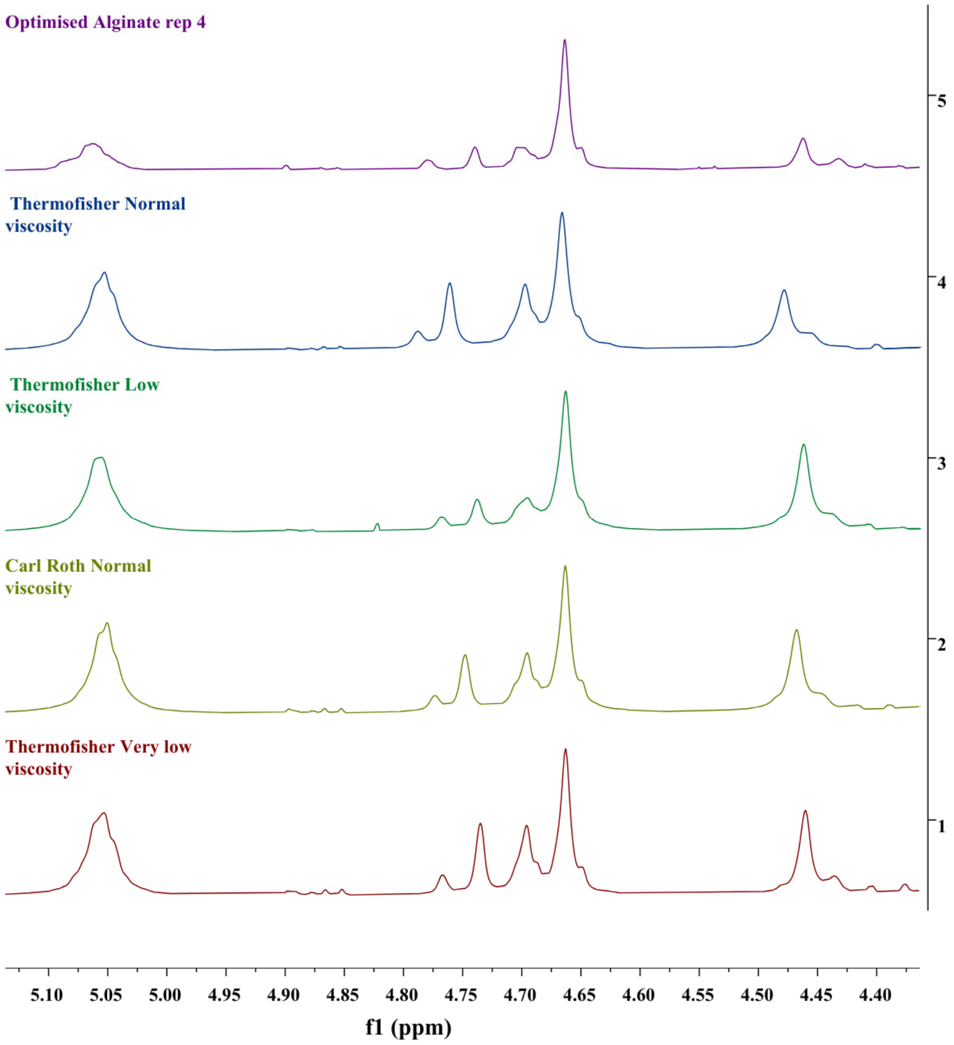

Figure S1: Stacked ^{1}H NMR spectra of the optimized extracted alginate (ALG) replicate four and the four commercial alginate standards. Spectra are shown in the anomeric proton region (approximately 4.35–5.15 ppm) and were used to determine the mannuronic acid to guluronic acid (M/G) ratio based on the characteristic resonances of the alginate monomer units.

Table S1: BBD experimental design and measured responses for the citric acid mechanochemical extraction of *Laminaria digitata* alginate, including extraction time, acid-to-algae ratio and blade speed, crude alginate yield, intrinsic viscosity, viscosity-average molar mass, mannuronic-to-guluronic acid ratio (M/G), protein content, ash content, total phenolic content.

Experiment	Time (min)	Acid/Algae	Speed (rpm)	Crude Yield (%)	Intrinsic Viscosity (dL g^{-1})	Mv (kDa)	M/G ratio	Protein (mg/g)	Ash Content (%)	Total Phenolic Content (mg GAE g^{-1})
**1**	10	2%	1100	13.33	5.5	261.4	1.17	1.34	17.87	6.68
**2**	60	2%	1100	18.72	10.61	509.7	1.06	1.72	16.54	6.78
**3**	10	4%	1100	20.61	9.55	458.0	1.35	1.22	17.57	7.98
**4**	60	4%	1100	10.55	13.14	633.4	1.59	1.41	18.16	5.08
**5**	10	3%	200	17.22	9.55	458.0	1.65	1.36	33.16	5.88
**6**	60	3%	200	21.11	9.57	459.0	1.34	1.15	15.62	5.88
**7**	10	3%	2000	17.33	9.32	446.8	1.77	1.39	33.06	5.48
**8**	60	3%	2000	18.88	9.9	475.0	1.39	1.41	17.48	6.48
**9**	30	2%	200	8.33	9.69	464.8	1.6	1.26	16.31	6.08
**10**	30	4%	200	7.88	10.92	524.8	1.79	1.39	18.06	10.08
**11**	30	2%	2000	8.05	7.08	337.9	1.13	1.43	16.36	8.78
**12**	30	4%	2000	9.27	12.45	599.6	1.43	1.39	16.32	9.48
**C(a)**	30	3%	1100	17.22	7.03	335.5	1.36	1.36	35.73	9.08

Table S2: M/G ratio and individual block fractions of the alginate extracted from each experiment in the BBD experimental design.

Experiment	M/G	F_G	F_M	F_{GG}	F_{MM}	F_{GM}	F_{GGG}	F_{MGM}	F_{GGM}
**1.**	1.17	0.46	0.54	0.26	0.58	0.20	0.18	0.16	0.08
**2.**	1.07	0.48	0.52	0.33	0.37	0.15	0.28	0.10	0.05
**3.**	1.35	0.43	0.57	0.21	0.36	0.22	0.06	0.06	0.15
**4.**	1.60	0.39	0.61	0.23	0.46	0.15	0.17	0.09	0.06
**5.**	1.65	0.38	0.62	0.16	0.41	0.22	0.04	0.10	0.11
**6.**	1.34	0.43	0.57	0.26	0.40	0.17	0.16	0.08	0.10
**7.**	1.77	0.36	0.64	0.18	0.46	0.18	0.07	0.08	0.10
**8.**	1.40	0.42	0.58	0.26	0.43	0.15	0.22	0.11	0.05
**9.**	1.61	0.38	0.62	0.22	0.46	0.16	0.17	0.11	0.05
**10.**	1.79	0.36	0.64	0.20	0.48	0.16	0.08	0.05	0.12
**11.**	1.14	0.46	0.54	0.29	0.37	0.17	0.21	0.09	0.08
**12.**	1.44	0.41	0.59	0.23	0.41	0.18	0.16	0.11	0.07
**C(a)**	1.35 ± 0.04	0.43± 0.01	0.57± 0.01	0.25± 0.01	0.40± 0.01	0.17± 0.00	0.19± 0.01	0.11± 0.01	0.06± 0.01

Table S3. Regression coefficients for the quadratic RSM model of alginate yield

Term	Estimate	Std. Error	t value	P value
(Intercept)	-40.07	8.29	-4.83	0.002
Time	-0.07	0.14	-0.52	0.620
Acid	36.06	4.87	7.40	<0.001
RPM	0.01	0.00	1.60	0.154
Time × Acid	-0.15	0.03	-4.89	0.002
Time × RPM	-0.00	0.00	-0.74	0.484
Acid × RPM	0.00	0.00	0.53	0.615
$Time^2$	0.01	0.00	6.57	<0.001
$Acid^2$	-5.20	0.77	-6.76	<0.001
RPM^2	-0.00	0.00	-3.07	0.018

Table S4. Regression coefficients for the quadratic RSM model of M/G ratio

Term	Estimate	Std. Error	t value	P value
(Intercept)	1.33	1.13	1.18	0.27
Time	-0.03	0.02	-1.40	0.20
Acid	0.93	0.82	1.13	0.29
RPM	-0.00	0.00	-1.72	0.12
Time × Acid	0.00	0.00	0.75	0.48
Time × RPM	-0.00	0.00	-0.15	0.89
Acid × RPM	0.00	0.00	1.68	0.13
$Time^2$	0.00	0.00	1.16	0.28
$Acid^2$	-0.25	0.16	-1.51	0.17
RPM^2	—	—	—	—

Table S5. Regression coefficients for the quadratic RSM model of Protein (mg g^{-1})

Term	Estimate	Std. Error	t value	P value
(Intercept)	1.46	0.62	2.35	0.05
Time	0.01	0.01	0.55	0.60
Acid	-0.22	0.37	-0.60	0.57
RPM	0.01	0.00	0.87	0.41
Time × Acid	-0.00	0.00	-1.00	0.35
Time × RPM	0.00	0.00	1.00	0.35
Acid × RPM	-0.000	0.00	-0.71	0.50
$Time^2$	0.00	0.00	0.03	0.98
$Acid^2$	0.05	0.00	0.87	0.41
RPM^2	-0.00	0.00	-0.75	0.48

Table S6. Regression coefficients for the quadratic RSM model of Total phenolic content (mg GAE g^{-1})

Term	Estimate	Std. Error	t value	P value
(Intercept)	-4.5567	8.14	-0.56	0.59
Time	0.1813	0.13	1.30	0.23
Acid	1.9836	4.82	0.41	0.69
RPM	0.0024	0.00	0.66	0.53
Time × Acid	-0.0145	0.03	-0.47	0.65
Time × RPM	0.000004	0.00	0.11	0.92
Acid × RPM	-0.000386	0.00	-0.44	0.67
$Time^2$	-0.0021	0.00	-1.61	0.15
$Acid^2$	-0.1435	0.76	-0.19	0.86

Table S7. Regression coefficients for the quadratic RSM model of Ash (%)

Term	Estimate	Std. Error	t value	P value
(Intercept)	-94.6172	22.54	-4.20	0.00
Time	0.2398	0.38	0.62	0.55
Acid	79.1139	13.37	5.92	0.00
RPM	0.0165	0.01	1.67	0.14
Time × Acid	0.0184	0.08	0.21	0.84
Time × RPM	0.000025	0.00	0.26	0.80
Acid × RPM	-0.000497	0.00	-0.21	0.84
$Time^2$	-0.0070	0.00	-1.97	0.09
$Acid^2$	-13.1313	2.11	-6.20	0.00
RPM^2	-0.000007	0.00	-2.75	0.03

Table S8. Intrinsic viscosity (dL g^{-1}) and viscosity average molar mass (kDa) for the referenced material and optimized crude alginate. Commercial alginate values represent single measurements, while optimized extracted alginate values represent mean ± standard deviation (n = 5).

**Material**	**Intrinsic viscosity (dL g^{-1})**	**Viscosity-average molar mass (kDa)**
Thermo Fisher very low viscosity	2.65	118.2
C. Roth normal viscosity	9.86	444.8
Thermo Fisher low viscosity	5.29	235.8
Thermo Fisher normal viscosity	13.59	617.1
Optimized extracted alginate	9.01 ± 0.99	406.3 ± 45.3

Table S9: Fractional and block sequence distribution of the optimized extracted alginate and commercially available alginate references, including the mannuronic-to-guluronic acid ratio (M/G) and the relative diad and triads of the F_G, F_M, F_{GG}, F_{MM}, F_{GM}, F_{GGG}, F_{MGM} and F_{GGM} sequence motifs. Values are presented as mean ± SE where applicable.

**Material**	**M/G**	**F_G**	**F_M**	**F_{GG}**	**F_{MM}**	**F_{GM}**	**F_{GGG}**	**F_{MGM}**	**F_{GGM}**
Optimized extracted alginate	1.99 ± 0.03	0.33 ± 0.00	0.67 ± 0.00	0.20 ± 0.00	0.53 ± 0.00	0.13 ± 0.00	0.15 ± 0.00	0.08 ± 0.00	0.05 ± 0.00
Thermo Fisher very low viscosity	1.20	0.46	0.54	0.27	0.36	0.18	0.23	0.14	0.04
C. Roth normal viscosity	1.12	0.47	0.53	0.33	0.38	0.15	0.29	0.11	0.04
Thermo Fisher low viscosity	1.13	0.47	0.53	0.31	0.37	0.16	0.27	0.13	0.03
Thermo Fisher normal viscosity	1.19	0.46	0.54	0.26	0.34	0.20	0.21	0.15	0.05

Table S10. Fiber diameter of wet-spun alginate fibers from optimized samples

Replicate number	Fiber diameter (μm)	Morphological note
1	265	Ribbed surface, uniform structure
2	201	Ribbed surface, slightly finer filament
3	182	Ribbed surface, most compact structure
4	269	Ribbed surface, minor longitudinal fold observed

Additional images:

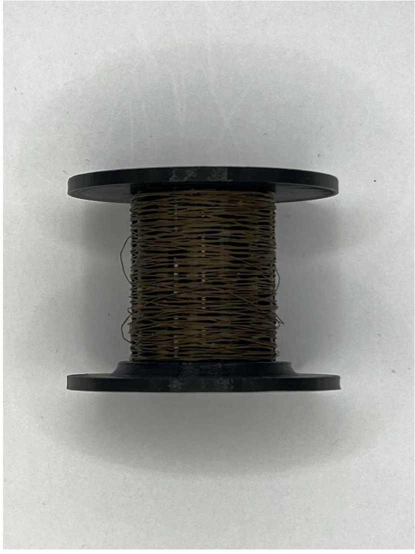

Spool sample
